# SARS-CoV-2 bioaerosol transmission in experimentally infected American mink

**DOI:** 10.1038/s41598-025-08111-1

**Published:** 2025-07-01

**Authors:** Rasmus Malmgren, Vinaya Venkat, Jenni Virtanen, Kristel Kegler, Thanakorn Niamsap, Lauri Kareinen, Olga Kivelä, Nina Atanasova, Pamela Österlund, Teemu Smura, Antti Sukura, Lara Dutra, Olli Vapalahti, Heli Nordgren, Ravi Kant, Tarja Sironen, Kirsi Aaltonen

**Affiliations:** 1 Molecular and Integrative Biosciences Research Programme, Faculty of Biological and Environmental Sciences, University of Helsinki, Viikinkaari 9, Helsinki, 00790 Finland; 2 Department of Veterinary Biosciences, Faculty of Veterinary Medicine, University of Helsinki, Helsinki, 00014 Finland; 3 Department of Virology, University of Helsinki, Helsinki, 00014 Finland; 4 Finnish Meteorological Institute, Erik Palménin Aukio 1, Helsinki, 00560 Finland; 5 Microbiology Unit, Finnish Institute for Health and Welfare, Helsinki, Finland; 6 Clinical Microbiology, Helsinki University Hospital, HUS Diagnostic Center, University of Helsinki, Helsinki, 00029 Finland; 7 Department of Tropical Parasitology, Institute of Maritime and Tropical Medicine, Medical University of Gdansk, Gdynia, 81-519 Poland

**Keywords:** SARS-CoV-2, Viral transmission

## Abstract

The SARS-CoV-2 BA.1 (Omicron) variant, which emerged in late 2021, is more transmissible than earlier variants but causes milder symptoms in humans. Mink farms, where animals are housed in close quarters, present a high risk for virus transmission and mutation, necessitating strict control measures due to documented cases of mink-to-human and human-to-mink transmission. Hence, we aimed to detect infectious airborne SARS-CoV-2 using BioSampler-air collectors and to investigate aerosol transmission between groups of American mink (*Neovison vison*). Two groups (male and female) were infected with the BA.1 variant, and samples were collected from aerosols, saliva, feces, and surfaces. The results indicated that infectious viruses were predominantly detected in aerosol samples over a three-day period in both groups. Surface, saliva, and fecal samples also showed potential for virus transmission. Notably, infectious viruses were cultivated from aerosol samples, confirming aerosol transmission among American mink. This study highlights the importance of immediate sample culturing to improve infectious virus detection and emphasizes the need for enhanced preventive measures on mink farms to mitigate the spread of viruses.

**Supplementary Information:**

The online version contains supplementary material available at 10.1038/s41598-025-08111-1.

## Introduction

SARS-CoV-2, a single-stranded RNA virus and the causative agent of the COVID-19 pandemic, is known to transmit among humans through the air via aerosols and droplets, and contaminated surfaces^1^. Since the emergence of the pandemic the virus has mutated and developed variants such as Alpha, Beta, Delta and Omicron variants. In this study, we focused on the more transmissible variant of SARS-CoV-2, BA.1 Omicron^2^, which has shown milder symptoms in humans^3,4^.

Besides humans, SARS-CoV-2 has been shown to infect various animals, including American mink (*Neovison vison*)^5,6^. American mink, though solitary in the wild, are co-housed in large numbers at fur farms, increasing the risk of virus transmission among the animals^7^. Such an environment serves as a reservoir for viral transmission^8^ and the accumulation of mutations, increasing the possibility of animal-to-human transmission^9,10^. Due to the open design of these farms, stray and wild animals, as well as birds, may encounter mink and their excreta, potentially carrying infections outside the farm^11,12^.

Cases of human-to-mink and mink-to-human transmission of SARS-CoV-2 have been documented^13–15^. Thus, as a biosafety measure, most infected farms resorted to mass culling of millions of animals^16^. To prevent future crises, a thorough investigation on the sources and transmission of virus infections needs to be conducted to help inform the measures taken during future outbreaks.

Some studies have found SARS-CoV-2 on the surfaces of mink farms using PCR and animal bedding, but the presence of infectious viruses have not been detected^6,12^. Additionally, SARS-CoV-2 is known to be transmitted via aerosols among humans^17–19^. The virus was found to be infectious in aerosols for up to 3 h in laboratory conditions^20^ but detecting infectious viruses from air samples in clinical and environmental settings has proven difficult^21,22^. Likely, this difficulty arises from the long delays between sample collection and culturing, as samples are often transported to distant laboratories for analysis.

This manuscript is a part of multiple publications on a study of American mink experimentally infected with SARS-CoV-2. In a previous publication, we proved that the Omicron variant, despite being known for mild infections in humans, can also infect mink^23^. In this manuscript, we focused on consistently capturing infectious viruses in aerosols collected around the experimentally infected mink, to provide evidence of aerosol transmission of SARS-CoV-2 at fur farms.

## Materials and methods

### Virus stock, cells lines and cell media

Virus stocks, cell lines, and cell media were used as previously published in Virtanen et al. (2022)^23^. SARS-CoV-2 BA.1 was acquired from the Finnish Institute of Health and Welfare ((original patient sample: hCoV-19/Finland/THL-202126660/2021, EPI_ISL_8768822 (Gisaid)). TMPRSS2-expressing VeroE6 (VE6T) cells were used for virus cultivation and were grown according to Rusanen et al. (2021)^24^.

### Experimental setup, animal infection and euthanasia

**Fig. 1:**
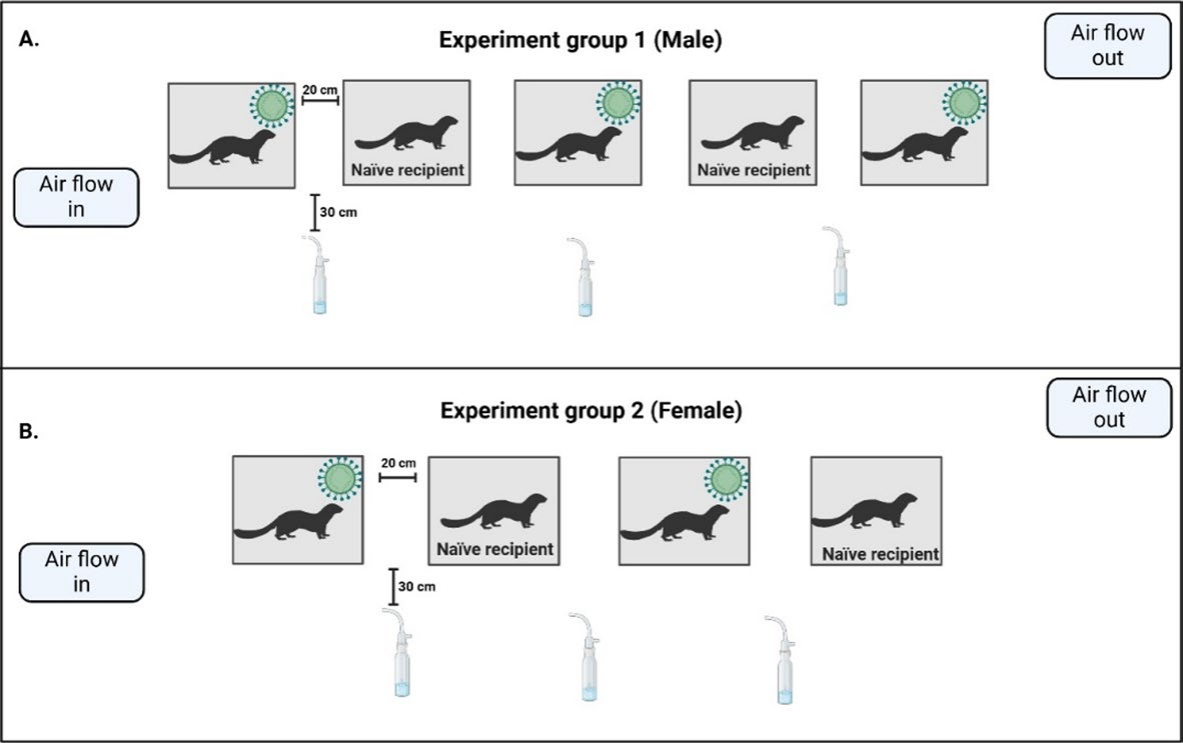
Experimental setup for male (A) and female (B) groups in the BSL3 facility. Metal bar enclosures were placed 10–20 cm apart on a table and the BioSamplers were placed 30 cm from the enclosures, at the height of the animals, to collect air samples from the area surrounding the mink. The samplers’ inlets were faced towards the table the enclosures were on. Inoculated animals are marked with virus illustrations and naïve recipients are as indicated in the figure. Figure created in Biorender (https://BioRender.com).

The mink were purchased from a commercial mink farm. Animals were acclimatized to the BSL3-laboratory room and custom-made enclosures for three days before infection. The enclosures were 84 × 76 × 58.4 cm metal bar cages, 10–20 cm apart, with a 26.2 × 31.5 × 40 cm closed metal nest with an open bar cage roof, containing hay as nesting material. The laboratory room was 59 m^2^ with ventilation flowing from left to right (Fig. 1) at a rate of approximately 242 l/s. Airflow in the room was turbulent or partially turbulent and could flow through animal enclosures. Animal waste was collected under the enclosures on a removable metal tray. Refer to animal enclosure setup in Fig. 1.

Before infection, animals were anesthetized using 30 µl of Ketaminol (100 mg/ml, Intervet, Netherlands) and Domitor (1 mg/ml, Orion Pharma, Finland). Three male and two female mink were nasally infected with 200 µl of SARS-CoV-2 BA.1 stock (pfu = 10^6^), as indicated in Fig. 1. Naïve recipient mink received PBS instead of the virus stock. Sedation was reversed using Revertor (5 mg/ml, Scanvet, Poland). One female mink had to be excluded from the study due to stress, resulting in only four animals in the female group. The nasally infected animals were euthanized in a CO_2_ chamher on 7 dpi (days post infection), while the recipient males and females were euthanized at 10 and 11 dpi respectively. The experimental infection, sedation and euthanasia of the American mink are described in more detail in Virtanen et al. (2022)^23^.

Clinical signs of nasal irritation, diarrhea, anorexia, cough, eye irritation and malaise were assessed daily by a veterinarian. A score of 0–3 was given for each clinical sign according to their severity. After euthanasia, histopathological signs of conchae, bronchiolitis, interstitial pneumonia, alveolar damage, syncytia, vascular lesions and pneumocyte II hyperplasia in the nasal cavity and lungs of the animals were given a score of 0–3 according to severity. Total scores were calculated to evaluate illness severity in male and female groups. Clinical sign total score was calculated for 0–10 dpi, as the male mink were only observed for 10 dpi.

Experiment 1 consisted of five males while experiment 2 consisted of four females. The experiments were conducted successively. Reversing of the sedation of the animals indicated the starting point of the experiment.

### Sample collection

Three air samples were collected simultaneously twice a day, before and after feeding, for 30 min using three 5 ml BioSamplers (SKC Inc., USA) positioned 30 cm from the enclosures, at the height of the animals. The samplers’ inlets faced the table the enclosures were on (Fig. 1). All samplers were operated with a single air pump, with individual airflows monitored using Mass Flowmeter 3063-devices (TSI Inc., USA). BioSamplers were thoroughly disinfected and washed with ethanol and MilliQ-water between collections. Aerosols were collected into 5 ml of minimal essential eagle’s medium (MEM, Sigma-Aldrich, USA) using a flow rate of 12.5 LPM. Amphotericin B (Gibco, USA) was added to MEM for the female group after some samples from the male group had fungal contaminations. Due to dehydration, aerosol samples were refilled with fresh media to 3 ml before analysis.

All animal and surface sample collection was done using Sigma Virocult^®^-swabs (MWE, UK). Saliva was collected as animals chewed on the swab, fecal samples collected from swabbing the feces, and surface samples collected using MEM dipped swabs swabbing a comprehensive surface area. Samples were collected in duplicates and post sampling the swabs were stored in 1 ml of MEM awaiting further processing.

One of the saliva and surface duplicates were transferred to cells for virus cultivation while the others were stored at -80 °C until PCR analysis.

### Virus cultivation

1 ml of samples were mixed with 2 ml of culture media and added to VE6T-cells on 6-well plates. Cells were incubated in 37 °C for 9 days or until cytopathic effect (CPE) could be detected. A 140 µl sample was taken from wells with CPE for RNA extraction to confirm SARS-CoV-2 as the causing agent. Extracted RNA was analyzed using PCR.

### PCR

Saliva and surface samples were extracted using the QIAamp Viral RNA Mini Kit (QIAGEN), while fecal and cell culture samples were extracted using QIAamp 96 Virus QIAcube HT kit (QIAGEN, off-board lysis). All samples were PCR tested for SARS-CoV-2 using Luna SARS-CoV-2 RT-qPCR Multiplex Assay Kit (NEB), targeting 2019-nCoV_N1 and 2019-nCoV_N2. Samples were positive (+) if they gave a signal with both probes, weak positive ((+)) if they only gave a signal with one probe, and negative (-) if they gave no signal. Cell culture samples were considered positive (+) if their Ct values were more than 5 cycles lower than the original non cell-cultured sample, possibly positive ((+)) if their Ct values were 1–5 cycles lower, and negative (-) if the Ct values were similar or higher.

### Statistical analysis

Statistical analysis was performed using Graphpad Prism 10.5.0. Correlation between variables was calculated using the two-tailed Pearson correlation test.

### Ethics statement

Experimental procedures were approved by the Animal Experimental Board of Finland (ESAVI/33259) and carried out accordingly. This study is performed in accordance with relevant guidelines and regulations. All methods are reported in accordance with ARRIVE guidelines.

## Results

### SARS-CoV-2 Omicron transmission among Mink

**Fig. 2:**
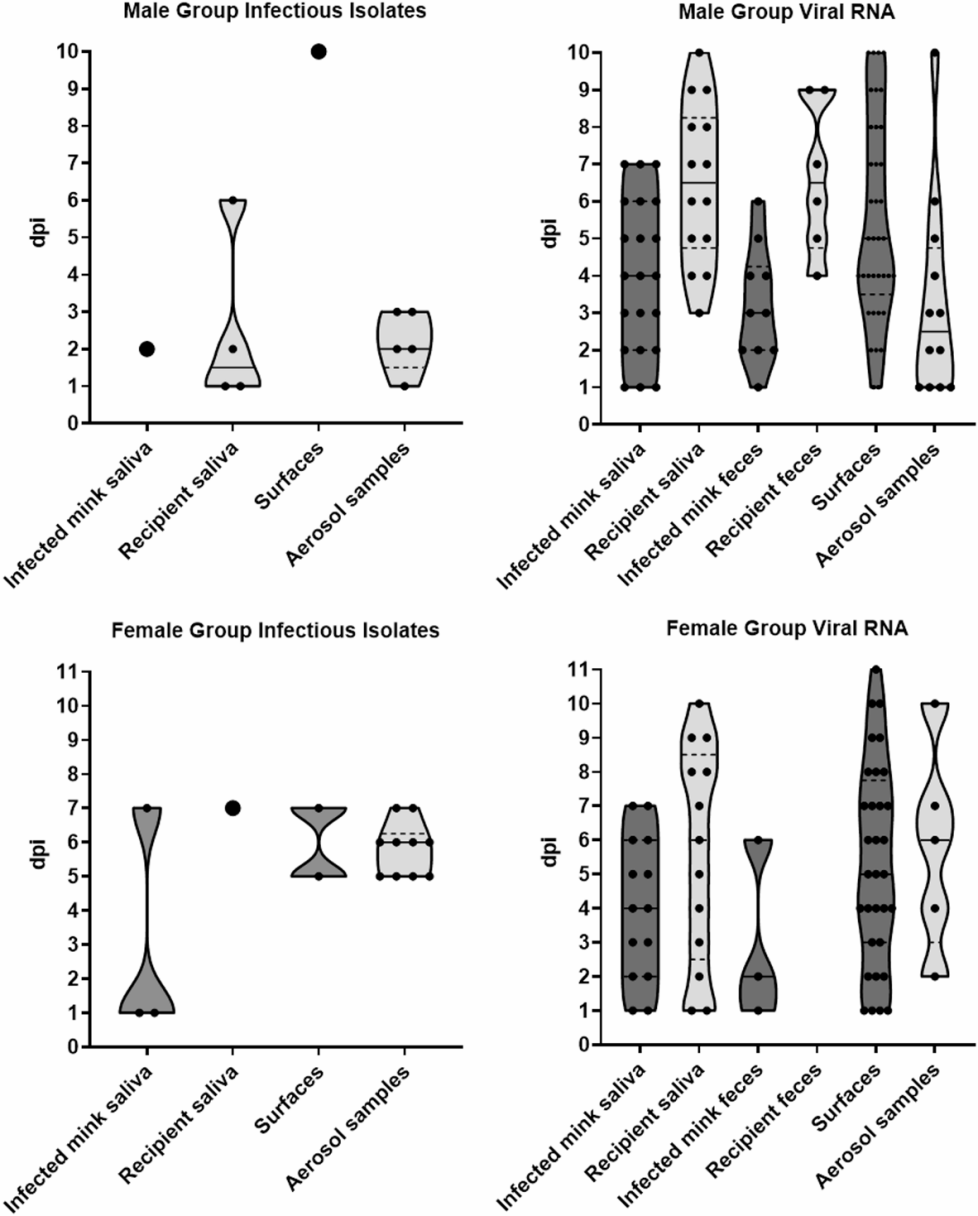
Positive cell culture and PCR findings. Infectious isolates represent cultivated virus samples, while viral RNA represents all viruses, infectious and inactive. The thickness of the graph describes the relative number of SARS-CoV-2 positive samples on each day post infection (dpi). Individual positive samples are represented with dots on the graphs. Positive sample medians are illustrated with full lines and quartiles with dashed lines. Negative or weakly positive samplesare not shown in the graph, but they are presented with all other sample data in Supplement Table 1, and 2. On day four, 10 surfaces were sampled instead of four (Supplement Table 1, and 2). Graphs created with GraphPad Prism 10.2.1.

Infectious viruses were detected from aerosol samples in both experiments (15/126 samples). For the males, infectious viruses were detected mostly on the first 3 days post infection (dpi) while in the female group they were detected later, mostly on 5–7 dpi (Fig. 2). PCR-positive aerosol samples were collected throughout the experiment from both groups (Fig. 2). Most sampled surfaces tested PCR-positive, however, infectious viruses were recovered only from 3/95 samples (Supplement Table 1, and 2). A few infectious samples were collected from animal saliva in both groups (9/77 samples), however, no infectious viruses were detected by culture in most samples (Fig. 2). In the male group, infectious viruses were detected in the saliva of infected animals only during 1–3 dpi, and in the recipient mink’s saliva already on 1 dpi. In the female group infectious viruses were observed on 1 and 7 dpi (Fig. 2). All saliva samples tested PCR-positive for the infected mink in both groups. The male recipient mink saliva was consistently PCR-positive from 3 dpi onwards, while only one of the female recipients was (Fig. 2). PCR tests from infected mink feces were variably positive for both groups, however, more so in the male group. In the recipient mink feces, PCR-positive results were observed only in the male group (Fig. 2).

The highest average viral load detected with PCR was in saliva samples (Ct = 29.6, 28.1; 31.3 in male and female groups respectively), followed by feces (Ct = 33.7, 33.3; 36.2), surface samples (Ct = 34.6, 31.7; 38.2) and air samples (Ct = 36.5, 37.1; 34.9). Viral loads in PCR-positive samples were higher on average in the male group than in the female group in all except aerosols samples.

SARS-CoV-2 was not detected from the original sample with PCR in many of the aerosol and saliva samples where infectious viruses were detected by cell culture (Supplement Table 1, and 2). Samples taken from all cell cultures exhibiting CPE and presented here as positive were confirmed to contain SARS-CoV-2 with PCR.

The average total scores for clinical signs on 0–10 dpi in male and female mink were 27.4 & 26.75 respectively, and for histopathological signs 13 & 10.75 (Supplement Table 3, and 4).

Statistical analysis showed no significant correlation between infectious virus detection and viral load indicated by observed Ct-values in saliva (*P* = 0.85) or aerosol samples (*P* = 0.44). Significant positive correlation was found between Ct-values and successful surface sample cell cultivation (*P* = 0.046; *r* = 0.17). No significant correlation was found between observed Ct-values from saliva and clinical symptom severity (*P* = 0.84) or histopathological symptom severity (*P* = 0.74).

## Discussion

Experimental studies conducted in ferrets^24^ and hamsters^26^ show the production of infectious SARS-CoV-2 containing aerosols, but this has not been demonstrated in American mink (*Neovison vison*). Our study bridges that gap, as we show that American mink infected with SARS-CoV-2 BA.1 (Omicron) variant can transmit viral aerosols from infected to healthy animals in a laboratory setting. We were able to come up with better aerosol sampling methods to capture these live viruses, which were not possible in previous studies. In our work, infectious viruses were detected primarily in aerosol samples, indicating that virus transmission among animals is likely to occur when animal enclosures are in close proximity.

Previous studies on viral bioaerosol transmission have had difficulties in detecting infectious viruses from bioaerosols^27,28^ or have only focused on RNA detection^29–31^. Additionally, while filter collectors have been the most used device for SARS-CoV-2 air sampling^32^, liquid-based collectors have been found to retain virus infectivity much better^33,34^. Here, we successfully detected infectious viruses in multiple bioaerosol samples by using 5 ml BioSamplers and culturing the samples immediately after collection. A pattern was observed in the aerosol samples, where both groups of mink had a three-day period when most of the infectious viruses were detected. Interestingly, this period happened in the first 1–3 dpi in the male group and later at 5–8 dpi in the female group. It is likely that the recipient male mink were infected already on 1 dpi, resulting in an earlier peak in infectious virus detection from aerosol samples. Additionally, SARS-CoV-2 has been reported to have more severe symptoms in males^35–37^, which could be a reason for earlier infection and detection of virus-containing aerosols. However, as this experiment only consisted of two groups of animals, more studies are needed to confirm these differences. Still, the three-day period observed here is much shorter than reported in humans^38,39^. Moreover, as not all samples where infectious viruses were detected by cell culture were PCR-positive from the original sample, the virus concentrations in aerosols were likely low. This is also supported by the high average Ct-values in the aerosol samples (Ct = 36.5).

Most samples containing infectious viruses were detected with aerosol collectors, suggesting that aerosol transmission is the main mode of transmission for SARS-CoV-2 BA.1 variant when animals are in separate enclosures. However, some infectious viruses were also detected from surface- and saliva samples, making surface and contact transmission also possible routes for infection. These differences in infectious virus detection could also be explained by the fact that saliva contains antiviral proteins that inactivate viruses residing in the mouth^40^. Moreover, due to the high ventilation in the room (242 l/s), the collected aerosols are likely freshly formed while viruses on sampled surfaces could have had more time to be inactivated. Collection method sensitivity could also affect these results; while Biosamplers have been found to retain virus infectivity well^33,34^, high initial Ct-values of < 30 in swab samples, like in the present study, have been reported to affect virus cell culturing negatively^41,42^. Here in contrast to previous studies^41,42^, statistical analysis showed no such correlation between observed Ct-values and successful cell cultivation in any sample type. A positive correlation was observed in surface samples, however, it is likely due to a low count of infectious samples (3/95), making the data not optimal for statistical analysis. Another possible reason for the lack of statistically significant correlation is most likely the observed Ct-values being high in all samples, and therefore virus loads being low, making infectious virus detection unreliable^41,42^. More studies are needed comparing aerosol and swab collection to understand whether the correlation of RNA load and successful virus cell culturing is similar for both sampling methods. Nevertheless, these findings highlight the importance of aerosols in SARS-CoV-2 transmission.

While the SARS-CoV-2 Omicron variant has been shown to survive on surfaces for multiple days^43^, we observed infectious viruses in only three surface samples. By contrast, most surface samples were PCR-positive, indicating high environmental contamination, however the longevity of virus viability appears to be brief. Additionally, it is important to acknowledge that the initial titer of the virus affects its survival^44^. Therefore, viruses originating from salivary bioaerosols or droplets might not survive as long on surfaces due to a low virus titer in saliva^45,46^.

SARS-CoV-2 was also variably detected in the feces of both infected and recipient mink with PCR. Previous studies have suggested that, if accessible, infected mink feces could spread SARS-CoV-2 to other wildlife and farm animals, such as cats^47^ or foxes^48^. This is also supported by findings of antibodies against SARS-CoV-2 in escaped mink around mink farms^49^, however, these infections could have also occurred before escaping. Additionally, although birds have not been observed to be susceptible to SARS-CoV-2^50,51^, they could act as mechanical vectors and transport the virus from the feces to other housing units or even other farms^52^. As farmed mink often defecate through their enclosures onto the ground below, it is accessible by these wild animals. This area should be restricted to prevent the spreading of viruses within and outside the farms.

Overall, more PCR-positive samples were collected during the male group [63% vs. 45% of collected samples, (Supplement Table 1, and 2)], suggesting that male mink have higher virus shedding than female mink. However, this difference was not observed in infectious virus detections [13% vs. 15%, (Supplement Table 1, and 2)]. Similar findings regarding viral RNA shedding have previously been made in humans^53–55^. This is also supported by the higher average total score in histopathological signs from the male mink nasal cavity and lungs compared to female mink (13; male vs. 10.75; female). However, we did not observe this difference in the total score of the clinical signs (27.4; male vs. 26.75; female). This sampling is however quite limited in size and a larger dataset is needed to observe significant differences between male and female mink illness severity when infected with the SARS-CoV-2 omicron variant. More studies are also needed with better infectious virus detection methods to better understand the differences in infectious virus shedding between males and females.

Our study demonstrated that American mink infected with the SARS-CoV-2 BA.1 variant produce aerosols containing infectious virus particles, capable of spreading the virus to nearby animals. We also found that culturing aerosol samples immediately after collection significantly improves the detection of infectious viruses, making diagnostics for both infected animals and humans more accurate. To prevent animal suffering and avoid mink farms becoming reservoirs for respiratory viruses, enhanced preventive measures and surveillance are essential. However, these measures can only be effective if the various modes of transmission, including aerosol spread, are thoroughly researched and understood.

## Electronic supplementary material

Below is the link to the electronic supplementary material.

Supplementary Material 1

Supplementary Material 2

## Data Availability

The datasets used and/or analysed during the current study available from the corresponding author on reasonable request.
