# Supplementary material for: SARS-CoV-2 bioaerosol transmission in experimentally infected American mink
Source: Sci Rep. 2025 Jul 1;15:22270. doi: 10.1038/s41598-025-08111-1 (PMC12217755; doi:10.1038/s41598-025-08111-1)
Supplement: Supplementary file 1 — Supplementary Material 1 [file 41598_2025_8111_MOESM1_ESM.docx]

**Supplement**

**Supplement Table 1.** Male group culture and PCR results. Culture samples are marked positive (+) (green) if Ct>5, tentatively positive ((+)) (yellow) if Ct=1-5, and negative (-) (red). PCR samples are marked positive (+) if both probes gave a signal, weak positive ((+)) if only one probe gave a signal, and negative (-). Samples marked “N/A” were not tested.

**Supplement Table 2.** Female group culture and PCR results. Culture samples are marked positive (+) (green) if Ct>5, tentatively positive ((+)) (yellow) if Ct=1-5, and negative (-) (red). PCR samples are marked positive (+) if both probes gave a signal, weak positive ((+)) if only one probe gave a signal, and negative (-). Samples marked “N/A” were not tested.

**Supplement table 3.** Clinical signs in infected and recipient mink scored 0-3 according to severity.

**Supplement table 4.** Histopathology findings of the nasal cavity and lungs of mink scored 0-3 according to severity.
